# Diverse Processes Drive the Origination and Maturation of an Array of Enhancers and Silencers During a Vast Evolutionary Timescale of a Bicistronic Gene

**DOI:** 10.3390/genes17050519

**Published:** 2026-04-28

**Authors:** Nicholas Delihas

**Affiliations:** Department of Microbiology and Immunology, Renaissance School of Medicine, Stony Brook University, Stony Brook, NY 11794, USA; nicholas.delihas@stonybrook.edu; Tel.: +1-631-286-9427

**Keywords:** evolution of enhancers, silencers, bicistronic gene formation, evolutionary fixation of gene sequence, cultivator gene model, spatiotemporal gene expression, *Alu* TEs in enhancers, high GC and CpG content

## Abstract

**Background/Objectives:** A central question in molecular genetics concerns how transcriptional regulatory sequences and de novo genes originate and reach evolutionary fixation. In this study, we utilize the human bicistronic gene *SMIM45* as a model to analyze the evolutionary trajectories of gene development. This locus comprises several functional units: three enhancers (one featuring an embedded silencer), an exonic silencer that partially overlaps an ORF, a highly conserved ancestral sequence encoding a 68 aa microprotein, and a human-specific de novo gene encoding a 107 aa protein expressed spatiotemporally in embryonic brain tissues. **Methods:** The alignment of gene sequences from different species was used to determine the evolutionary development of enhancers and silencers, and the development of the exonic silencer was determined through application of the cultivator model and assessment of nearest-neighbor bases. **Results**: We identify significant disparities in formation mechanisms; for example, the *LOC127896430* NANOG hESC enhancer originated simply via two *Alu* insertions that constitute the enhancer. In contrast, the exonic silencer (a segment of the *LOC130067579* ATAC-STARR-seq lymphoblastoid silent region 13815)—a distinct, novel type of silencer—originated from a combination of diverse mechanisms, including a “cultivator gene” process of base pair fixation, consistent with the cultivator model proposed by Li Zhao and coworkers. **Conclusions:** *SMIM45* exemplifies novel development mechanisms occurring over hundreds of millions of years, culminating in the birth of a human-specific, de novo 107 aa cistron. The associated complex of enhancers and silencers suggests intricate regulation of the 107 aa protein in fetal brain tissues.

## 1. Introduction

Although bicistronic genes are uncommon in eukaryotes, they are being revealed more frequently using improved detection techniques [1,2,3]. These genes form a heterogeneous group with diverse mechanisms for RNA transcription and protein expression. For instance, some utilize alternative RNA transcript isoforms, such as those expressed spatiotemporally in hippocampal neurons [4], while others employ leaky scanning from an internal ribosome entry site (IRES) [5] or an upstream open reading frame (uORF) that inhibits cap-dependent translation [6]. *SMIM45* is also a bicistronic gene, encoding both an ancient 68 aa microprotein and a human-specific, de novo 107 aa protein [7]. Notably, while the 68 aa microprotein is expressed in somatic tissues, the 107 aa protein is expressed spatiotemporally in embryonic brain tissues [8,9]. A significant feature of *SMIM45* is that it contains an array of enhancers and silencers that likely regulate the transcription of the 107 aa cistron from its promoter, potentially representing a distinct transcriptional/translational mechanism for a bicistronic gene. This distinguishes the process from protein expression from a bicistronic transcript that relates to the above-mentioned processes. Thousands of bicistronic genes are yet to be characterized; notably, Raj et al. [10] identified over 2000 of them in human gene analyses. Given the large number of uncharacterized genes, *SMIM45* may represent a new class of bicistronic genes.

Enhancers and silencers are short regulatory sequences found abundantly throughout the human genome [11], which bind transcription factors and can function by regulating cell-specific transcription of genes in embryonic tissues [12,13]. Super-enhancers refer to multiple enhancers present in a genomic locus that ensure gene expression in specific tissues [14,15,16,17], while super-silencers denote the presence of two of more silencers in a gene locus that act together to provide strong signals for repression of gene expression, whereby repression is dependent on the locus’ high CpG content [18,19]. As functional data are not yet available, we have not termed the array of enhancers and silencers as super-enhancers/super-silencers; however, their presence lends credence to these terms.

Due to the regulatory elements present in *SMIM45*, the gene is well suited to an analysis of the evolutionary formation of enhancers and silencers. In this paper, we analyze the mechanisms of origination and the timeline of the appearance and completion of the enhancers and silencers during evolution. The study shows that regulatory elements formed through diverse mechanisms, highlighting the development of *SMIM45* via the continuous birth of functional elements over ~400 million years. In particular, our analysis reveals that the short, exonic silencer (a segment of the silencer *LOC130067579* ATAC-STARR-seq lymphoblastoid silent region 13815), which overlaps the C-terminal sequence of the 68 aa protein gene, originated and matured through a combination of distinct molecular processes. This silencer is unusual as it spans both a gene promoter and an ORF, and appears to be unique among known silencing elements. Comparisons are also made with known properties of silencers and enhancers, providing insight into how the expression of the 107 aa cistron may be regulated.

Emera et al. [20] investigated the evolution of enhancers, introducing a model of proto-enhancers as small, early developmental sequences that serve as nucleation sites for further development. We address the role of proto-enhancers/proto-silencers in *SMIM45* development, but note that Emera et al.’s model aligns with the previously described evolution of the 107 aa protein cistron. Here, development proceeds via the initial formation of a proto-gene, a short amino acid (aa) sequence called an early developmental sequence [7]. In this framework, the proto-gene originates in ancient species and matures through the contiguous fixation of nearest-neighbor bases of the original as well as secondary proto-genes.

### Background on SMIM45

**Figure 1:**
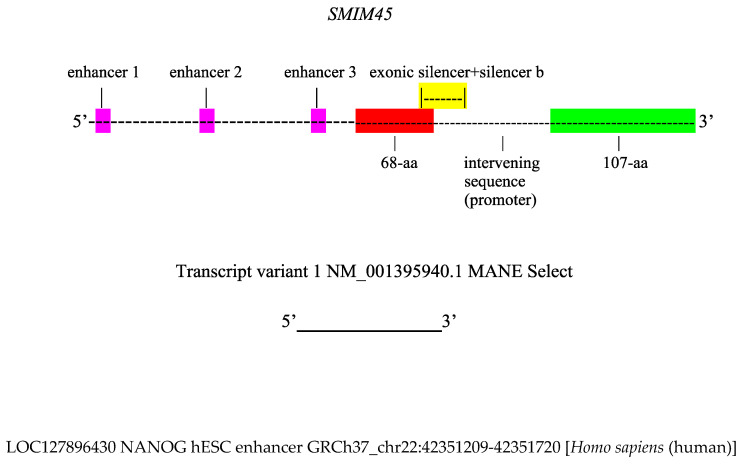
The*SMIM45* gene is 11,991 bps in length and contains three enhancers (marked in pink), the 68 aa protein ORF (in red), *LOC130067579* ATAC-STARR-seq lymphoblastoid silent region 13815 (the exonic silencer and silencer b) (in yellow), and the 107 aa protein ORF (in green). Enhancer designations: Enhancer 1, *LOC130067578* ATAC-STARR-seq lymphoblastoid active region 19151; Enhancer 2, *LOC127896429* H3K4me1 hESC enhancer GRCh37_chr22:42346983-42347610 *Homo sapiens*, which also contains an embedded silencer, *LOC127896429* silent region_13814; Enhancer 3, *LOC127896430* NANOG enhancer GRCh37_chr22:42351209-42351720 *H. sapiens.* The genomic distance between enhancer 1 and enhancer 2 is 3760 bps; between enhancer 2 and enhancer 3, it is 3599 bps. The exonic silencer is the segment of the transcriptional silencer *LOC130067579,* ATACSTARR-seq lymphoblastoid silent region 13815, that overlaps the 68 aa sequence by 38 bases. Silencer b is the segment of the silencer that overlaps the intervening promoter sequence by 192 bases. The yellow highlighted segment denotes the entire *LOC130067579* length. The promoter is in the intervening sequence and is −133 bp upstream of the 107 aa transcriptional start site. Transcript variant 1 is 1540 nt in length and encompasses the *SMIM45* sequence from the 68 aa to the end of the 107 aa sequence. It includes the exonic silencer but no enhancer sequences; however, the intron of processed transcript variant 1 contains the enhancer 3 NANOG hESC. Transcript variant 1 is termed as an MANE Select transcript, as it is annotated in both the NCBI and *Ensembl* databases with agreement on 5′ end start sites. The 13 other transcript variants also contain the sequence from the 68 aa to the end of the 107 aa sequence, and several carry enhancer 1, ATAC-STARR-seq lymphoblastoid active region 19151, in their exons.

Given the complex nature of the *SMIM45* gene and its regulatory landscape, a comprehensive background is provided. The *SMIM45* ultra-conserved 68 aa microprotein cistron is a pre-existing ancient gene that can be considered as a cultivator gene. Lee et al. [21] presented a model to explain the origination of regulatory elements and de novo genes based on a proposed cultivator gene: a pre-existing gene that enables the fixation of DNA sequences. On the 3′ side is a de novo 107 aa protein cistron found only in humans (Figure 1). The two *SMIM45* cistrons are linked to and separated by a transcriptional silencer, *LOC130067579,* an ATACSTARR-seq lymphoblastoid silent region 13815 [22], whose sequence partly overlaps with the 68 aa C-terminal end. We term this overlapping sequence as an exonic silencer. The remaining segment of the silencer, termed here as silencer b, resides in the intervening sequence (Figure 1). Given that enhancer sequences overlapping coding regions are designated as exonic enhancers [22], we term the similarly situated silencer sequence as an exonic silencer. About one-fourth of all protein genes contain enhancers [23], and an analysis of silencer regions identified in HepG2 cells reveals that about 4% of silencers are found in exons [24]. There appears to be no information on silencers that partially overlap open reading frames (ORFs). The enhancers are designated as enhancers 1–3 and are approximately evenly spaced in the *SMIM45* locus by ~3–4 kbps (Figure 1). Thus, the *SMIM45* gene and its RNA transcripts have complex arrangements, with transcripts carrying different enhancer sequences in exons or introns (Figure 1).

The initial study by An et al. [25] that described spatiotemporal expression of the *SMIM45* 107 aa protein was partially compromised; the annotation by the *Ensembl* and GenBank data bases for the *SMIM45* locus had not yet been updated from the previous annotation as a lincRNA gene, and detection of a protein was not originally reported. These issues have now been updated and rectified. With the use of human embryonic cells, the expression of a protein from the 107 aa cistron was shown using multiple experimental techniques. Using human fetal brain cells, Chuan-Yun Li and co-workers [8] reported a protein product from the 107 aa ORF using mass spectrometry and Western blot analysis via a specific polyclonal antibody. One of the peptides found, GSGLELVR, represents part of the early developmental proto-gene sequence formed during evolution of the 107 aa protein [7]. Immunochemistry analyses indicate expression in the human cerebral cortex and human cortical organoids grown from human embryonic stem cells. In addition, translation from the 107 aa ORF was detected through analysis of public Ribo-Seq datasets, where ribosome footprint signals were found from cortical organoids grown from human embryonic stem cells [9]. In other independent gene expression studies, an analysis of total RNA from various human tissues revealed RNA expression from *SMIM45* in fetal brain tissues (NCBI BioProject: PRJNA280600) [https://www.ncbi.nlm.nih.gov/gene/339674, (accessed on 12 April 2025)]. However, a 107 aa cistron transcript has not yet been isolated.

Table 1 lists the enhancers found in the *SMIM45* locus, together with several known properties of related enhancers. All three show functions that can pertain to regulation of the expression of the 107 aa protein; for example, Enhancer-2-related enhancers can act as both enhancers and silencers of gene expression, which matches the spatiotemporal expression of the 107 aa protein. Translation of the 107 aa protein was detected in the human embryonic cerebral cortex and human cortical organoids [8], where embryonic cerebral cortex tissues consist of proliferating stem/progenitor cells [26]. Enhancer-3-related enhancers bind transcription factor NANOG, which is known to activate several gene promoters in embryonic stem cells [27].

## 2. Results

### 2.1. Evolution of Exonic Silencer: Cultivator Fixes Bases in Silencer

The overlapping exonic silencer sequence has dual functions: it encodes the C-terminal end aa sequence of the 68 aa protein and forms a part of the silencer *LOC130067579*. We investigated how initial bases of the exonic silencer were formed and fixed during the early evolutionary stages of development and searched for the earliest living species that carry the *SMIM45* sequence; which, according to the available data bank annotations in *Ensembl*, is the elephant shark (*Callorhinchus milii*), whose age of divergence is ~435 Mya. There is no information on earlier animal species that may contain the *SMIM45* locus or its flanking genes; e.g., sponges, which are members of the phylum Porifera.

**Figure 2:**
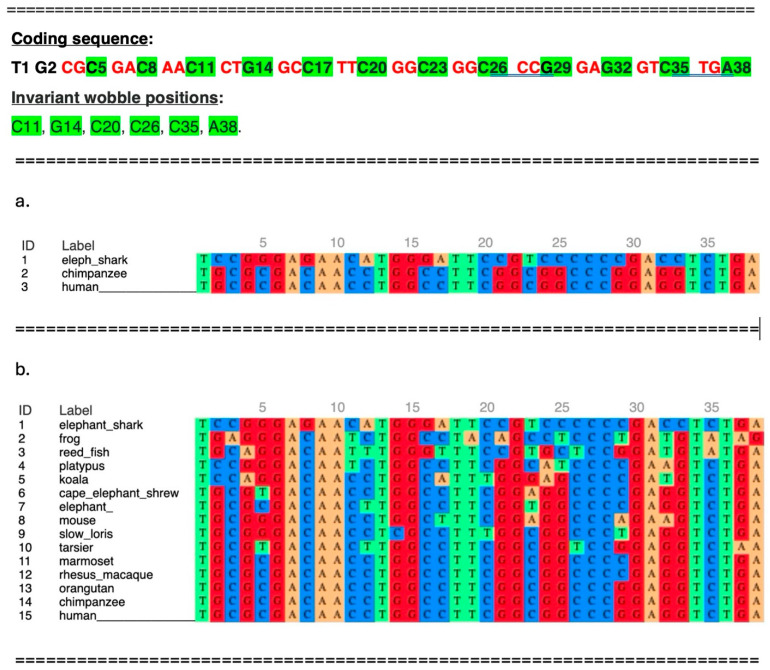
Alignment of the human exonic silencer sequence with sequences from other species. Top: The C-terminal 68 aa coding sequence; the first and second codon positions are highlighted in red, and the wobble positions in green. Invariant wobble positions are also highlighted in green. (**a**) Alignment of the human and chimp sequences with that of the elephant shark. (**b**) The first codon begins with the third base, CGC. The alignment of base sequences homologous to exonic silencer from ancestral species with that of the sequence of human exonic silencer. The order of alignment of species 1–15 is by age of evolutionary appearance. The alignment is by MAFFT (https://mafft.cbrc.jp/alignment/server/index.html, accessed on 15 December 2025).

A comparison of the exonic silencer sequence present in the elephant shark compared with that of the human sequence shows that 25 out of 38 bases (66%) are identical (Figure 2a). These bases were likely fixed in an ancestor of the elephant shark. Thus, much of the exonic silencer sequence is of ancient origins of over 435 Mya. In addition, 24 bases are fixed because they are in either the first or second position of a codon (Figure 2, top).

A change in bases of codons can result in a change in the aa sequence of the 68 aa C-terminal end. For example, a change in the invariant G6 to C6 translates to the aa sequence RHNLAFGGPEV; meanwhile, the human aa sequence is RDNLAFGGPEV. Thus, 24 bases, which also form the sequence of the exonic silencer, are fixed by the cultivator-68 aa sequence and by natural selection. This is consistent with and supports the cultivator model of Lee et al. [21].

Located within the 38 base silencer/68 aa sequence are 12 wobble bases (Figure 2, top, highlighted in green). Six of these twelve wobble bases, plus a seventh, T1, which is outside the ORF, are invariant between the elephant shark and humans (Figure 2a and top).

Because these DNA bases do not dictate amino acid fidelity, they represent a specialized conservation within the exonic silencer, likely fixed in an ancestor to the elephant shark. This raises an important question regarding the mechanism of origination and fixation of these seven invariant bases. However, this currently cannot be answered, as sequence analyses of *SMIM45* or its neighbor genes (*CENPM* and *SEPTIN3)* from species ancestral to the elephant shark, such as lamprey, sea star, or sponges, are currently not possible as these genes have not yet been annotated within the available genome assemblies.

**Figure 3:**
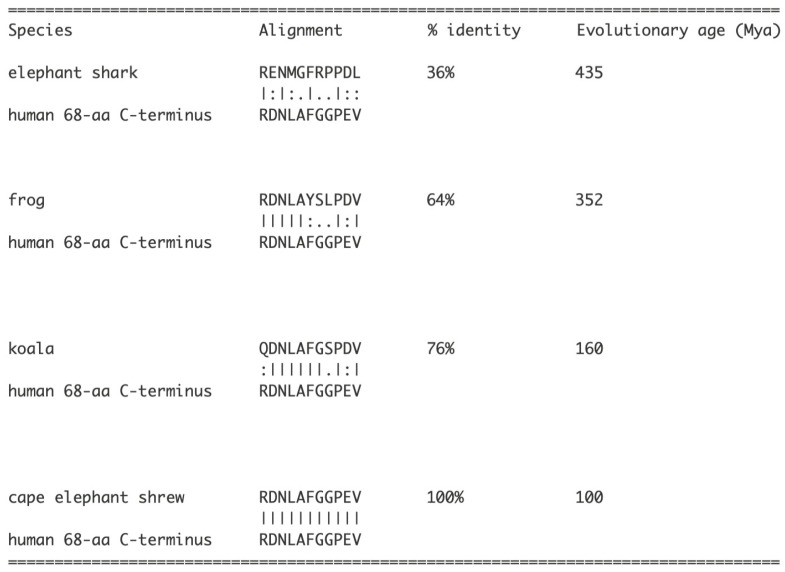
Translated aa sequence from the 38 nt sequence exonic silencer from different species and the percent identities. The homologous aa sequences from the mouse and primates are identical to that of the human. The ExPASy Translational tool (Swiss Institute of Bioinformatics, SIB, https://web.expasy.org/translate/, accessed on 15 December 2025) was used for the translation of nucleotide sequences. The aa sequences represent 5′ 3′ Frame 3 translations; evolutionary ages are for reference. Alignment was with EMBOSS Needle.

Figure 2b shows the multiple sequence alignment of the 38 base exonic silencer in humans with that of the homologous sequences from 14 species, demonstrating that the exonic silencer sequence was evolutionarily completed in the marmoset (a New World primate of the Platyrrhini parvorder) approximately 40 Mya. To determine when the amino-terminal end of the 68 aa ORF aa sequence was completed, 38 nt sequences from different species were translated. Figure 3 shows that the C-terminal end of the 68 aa ORF is completed in the Afrotheria clade.

The completion of the 68 aa C-terminal sequence within the Afrotheria clade is chronologically linked to the emergence of silencer b, the regulatory segment located in the intervening region (see Figure 1) that was previously shown to have originated in the Afrotheria clade [7]. Given this chronological linkage, we suggest that the fixed/invariant exonic bases were potentially key in the initial formation of silencer b, though via an unidentified cis-acting mechanism.

### 2.2. Evolution of Exonic Silencer: Further Development via Biased Mutations at Nearest-Neighbor Invariant Bases

We address the evolutionary expansion of the exonic silencer sequence, analyzing mutations that occur on the 5′ and 3′ sides of invariant bases. Thirteen bases are considered (Table 2), and invariant positions in codons where neighbor codon bases are fixed by natural selection are excluded. During evolution, nearest-neighbor mutations that produce G and C bases occurred almost entirely (12/13) (Table 2), suggesting that invariant bases and the sequence context steer mutational pressure toward GC accumulation within the exonic silencer. In other studies, it has been shown that biased nearest-neighbor mutations that occur during evolution depend on the identity of neighboring bases [31]. Although the small sample size precludes formal statistical analysis, the data show that mutations in nearest neighbors of invariant bases increase the GC and CpG content of the exonic silencer locus. The exonic silencer has a 70% GC content and contains five CpG dinucleotides, with three CpG sites contributed by nearest-neighbor bias. Silencers have been shown to repress gene expression in CpG-rich regions [18], which may pertain to repression of the expression of 107 aa cistron in somatic tissues. The mutations that occur at neighboring bases of invariant bases result in the total completion of the exonic silencer sequence present in humans.

Thus, the exonic silencer is formed through a combination of processes: codon-fixed bases (via the cultivator model), GC bias at nearest-neighbor invariant bases, and invariant wobble bases that were formed by unknown means and in unknown ancient species. The invariant wobble base analysis updates the previous estimate of ~352 Mya to ~435 Mya, which was based on synteny and sequence similarity [7].

### 2.3. Silencer B (Segment of Silencer LOC130067579 Within the Promoter): Detection of Species-Specific Sequences

Silencer b originated de novo within the Afrotheria clade by a pathway and at a different time than the exonic silencer [7]. Here, we show that its sequence was completed in the chimpanzee ~6 Mya (Figure S1). Four point mutations distinguish the chimpanzee/human sequence from that of the gorilla, which contrasts with completion of the exonic silencer in the New World primates ~40 Mya. Although the exonic silencer and silencer b differ significantly in their mechanisms and time frame of origin, both constitute the sequence of silencer *LOC13006757* and function within this context.

**Figure 4:**
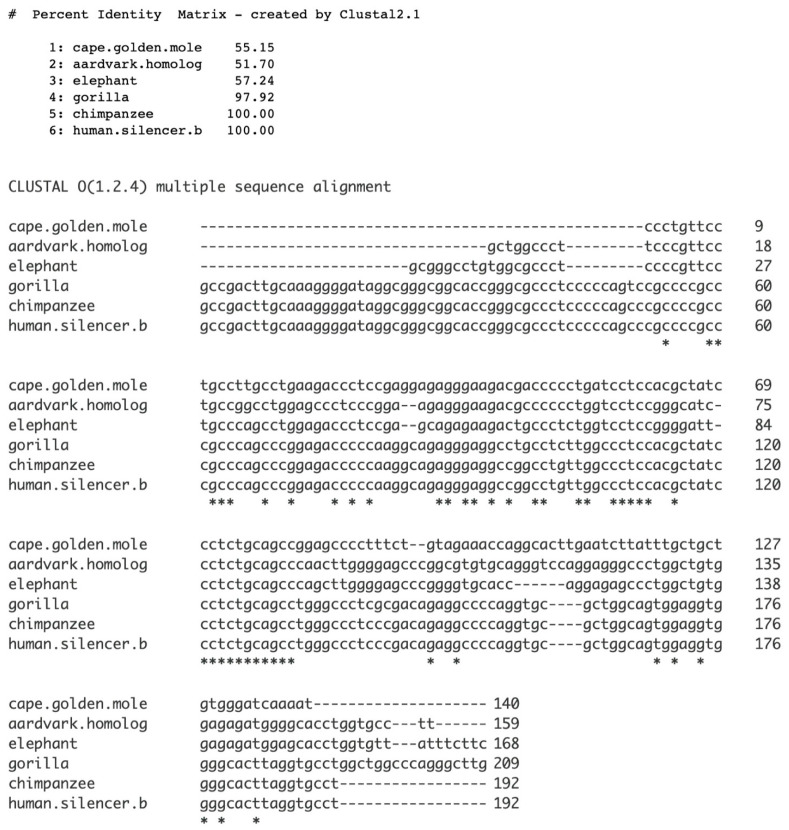
Alignment of silencer b with homologous sequences from representative Afrotheria and Great Apes species. Base positions 121–131 are totally conserved. Clustal Omega multiple sequence alignment (MSA) was used.

**Figure 5:**
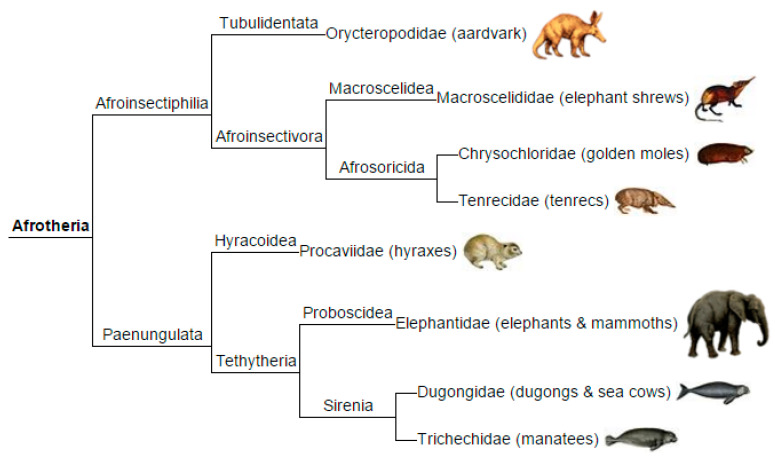
Phylogeny of the Afrotheria clade. Illustration is from WikiCommons and is in the public domain.

As silencer b originated de novo, we searched for a proto-silencer b sequence. Figure 4 shows an alignment of silencer b with orthologous sequences from the Afrotheria and the Great Apes. The longest sequence found to be invariant is cctctgcagcc (Figure 4). This 11 base sequence is 100% conserved across the deeply divergent mammalian lineages (Afrotheria to Primates, approximately 90 million years of separation). Furthermore, there is a synteny with the 68 aa bp sequence. Such high levels of conservation point to strong purifying selection and suggest that this sequence is likely a proto-silencer. As mentioned before, the fixed/invariant bases within the exonic silencer might have served a role by facilitating the formation of silencer b, with the necessary information for this process stored directly within those bases. However, it is unknown how fixed exonic silencer bases may have initiated the proto-silencer b sequence, despite their previously mentioned suggested importance. For reference, the Afrotheria phylogeny is shown in Figure 5.

Alignment of the silencer sequence with homologs from Afrotheria, Old World monkeys, and Great Apes reveals that a repeat, gcccgcccc, was formed in the Great Ape *SMIM45* locus during evolution (Figure S2), which is part of a longer repeat: gcccgccccgcccgccc. This indicates evolutionary pressure to increase the GC and CpG content of silencer b in the Great Apes. Silencer b contains 13 copies of CpG which, together with the 5 CpG dinucleotides present in the exonic silencer, results in 18 copies of CpG in the silencer *LOC130067579,* ATACSTARR-seq lymphoblastoid silent region 13815. This is consistent with the repression of gene expression dependent on the high CpG content of a locus [18,19].

### 2.4. Enhancer 1: Sequence Origin and Completion

**Figure 6:**
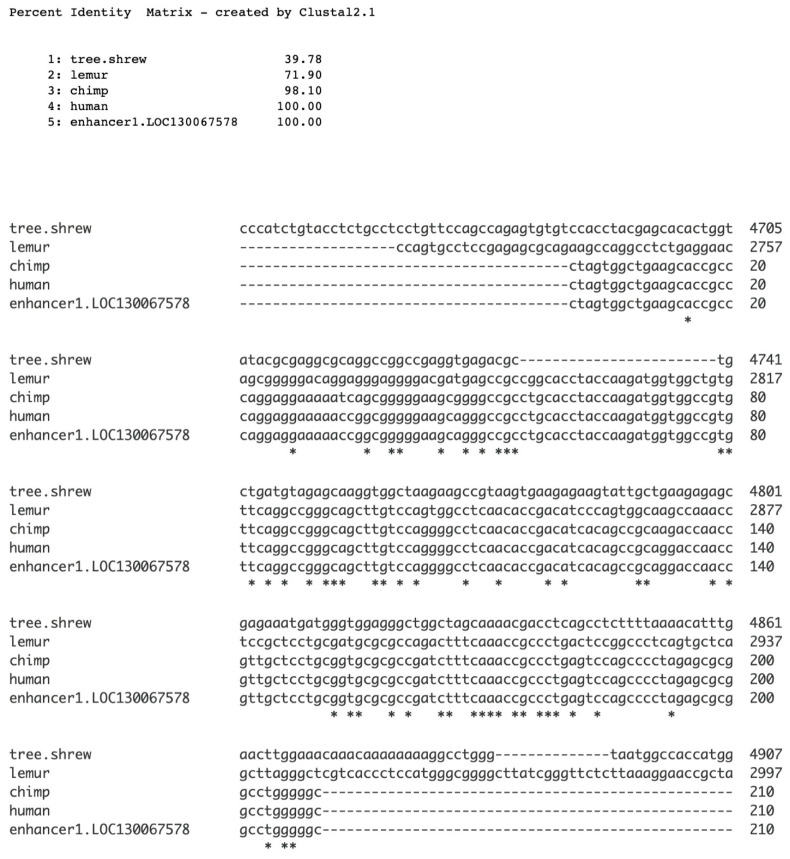
Alignment of enhancer 1 with genomic sequences of the tree shrew (*Tupaia chinensis*, Chinese tree shrew) and the lemur (*Lemur catta*, ring-tailed lemur). Both species are the closest living relatives to primates. Clustal Omega multiple sequence alignment (MSA) was used for alignment.

Enhancer 1 is situated eight base pairs from the 5′ start of the *SMIM45* gene and also resides within the exons of four transcript variants. Here, we investigate its evolutionary origin and sequence completion. Alignment with homologous regions in other primate species and in close primate relatives suggests that enhancer 1 originated de novo. The lemur sequence displays significant similarity with enhancer 1 (78%), and the tree shrew—also a close relative to the primates—shows no significant identity (40%) (Figure 6), while their age of divergence from a common ancestor is ~63 Mya and ~68 Mya, respectively. The data suggest that enhancer 1 originated in an extinct early stem-lineage of the lemur more than 60 Mya. It was not possible to identify a putative proto-enhancer 1, as sequence alignments with the tree shrew could not be made with accurate synteny. For reference, a phylogenetic tree of the primates is given in Figure S3.

**Figure 7:**
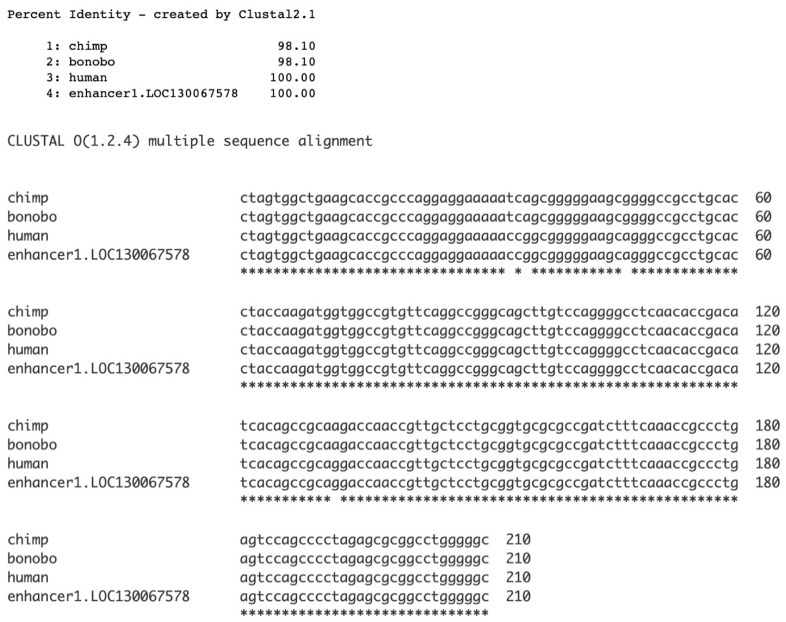
Completion of enhancer 1 sequence in humans. Clustal Omega multiple sequence alignment (MSA) was used for alignment.

Additionally, the species for which the enhancer 1 sequence was finalized was investigated. Figure 7 shows that there are four point mutations that distinguish the human from the two chimpanzee sequences. Enhancer 1 was thus completed in the human genome.

### 2.5. Enhancer 2, LOC127896429 H3K4me1hesc Enhancer GRCh37_chr22:42346983-42347610

**Figure 8:**
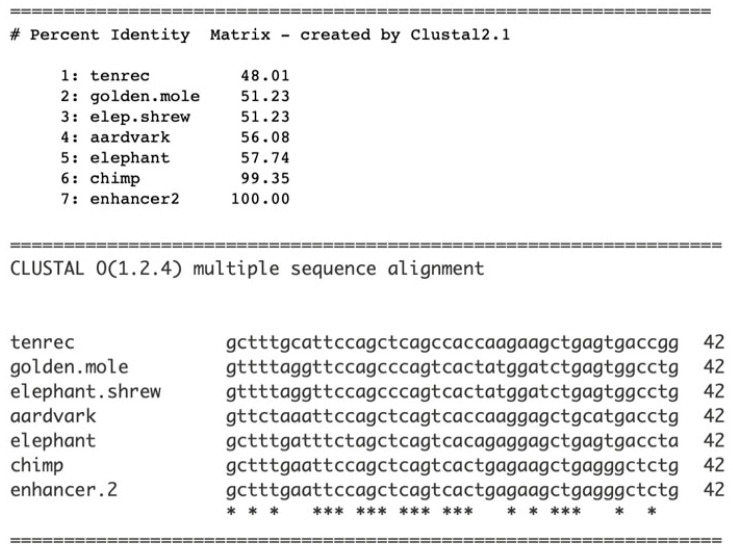
Homologous sequences from the Afrotheria clade were aligned with sequences of the chimpanzee and a segment of the enhancer 2 sequence that displays high conservation. Top, the percent identities. Bottom, a segment of enhancer 2 aligned with homologous sequences. Clustal Omega multiple sequence alignment (MSA) was used.

The evolutionary origination and completion of enhancer 2 was investigated. Similarly to that of enhancer 1, enhancer 2 originated de novo. Sequences from five members of the Afrotheria clade were aligned with those of the chimpanzee and the human enhancer 2. The percent identities show that sequences from several Afrotheria species approach the “twilight zone” of significance, suggesting origination from the Afrotheria clade (Figure 8, top), where the data suggest that enhancer 2 originated specifically within the clade’s Afroinsectivora branch (Figure 5). The koala (*Phascolarctos cinereus*) lineage separated from the ancestor of Afrotheria before the Afrotheria separated from other placental mammals, and its genomic sequence does not significantly align with enhancer 2 (45% identity). This is consistent with the determination that enhancer 2 originated in the Afrotheria clade, ~100 Mya. One segment of enhancer 2 displays invariance, _17_cagtcac_23_, with the exception of the tenrec sequence (Figure 8, bottom); this sequence may be a candidate for a proto-enhancer 2 as the 8-base sequence is under purifying selection, over ~90 millions of years of separation.

An alignment of the chimpanzee and human *SMIM45* sequences with that of the enhancer 2 reveals two bp deletions and three point mutations that distinguish the human sequence from that of the chimpanzee (Figure S4). Therefore, enhancer 2 is identified as human-specific. Nevertheless, similar to the mutations in enhancer 1, the functional impact of these mutations remains unknown. The completion of the embedded silencer, *LOC127896429* silent region_13814, was also determined. The sequence was completed in the chimpanzee (Figure S5) and therefore is not human-specific, contrasting with the completion of enhancer 2.

### 2.6. Enhancer 3, LOC127896430 NANOG Enhancer GRCh37_chr22:42351209-42351720 Homo sapiens: Birth by Alu Insertions 

**Figure 9:**
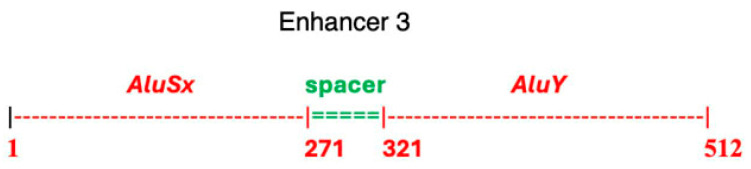
Schematic of components of human enhancer 3. *Alu* TEs and the spacer sizes were determined using RepeatMasker [37].

Branco and Chuong [32] have provided examples of how transposable elements can drive regulatory innovation. Functioning in gene regulation, many enhancers contain *Alu* transposable elements that modulate target gene expression through promoter interactions [33,34,35]. Nearly half of *Alu* elements present in the human genome are in introns, where they regulate gene transcription in specific tissues [36]. In accordance with this, enhancer 3 (*LOC127896430* NANOG hESC enhancer) contains two *Alu* elements (Figure 9). In *SMIM45* RNA transcripts, enhancer 3 resides in the intron of *SMIM45* RNA transcript variant 1, NM_001395940. Given its intron location, enhancer 3 may participate in the regulation of the 107 aa cistron in embryonic brain tissues.

**Figure 10:**
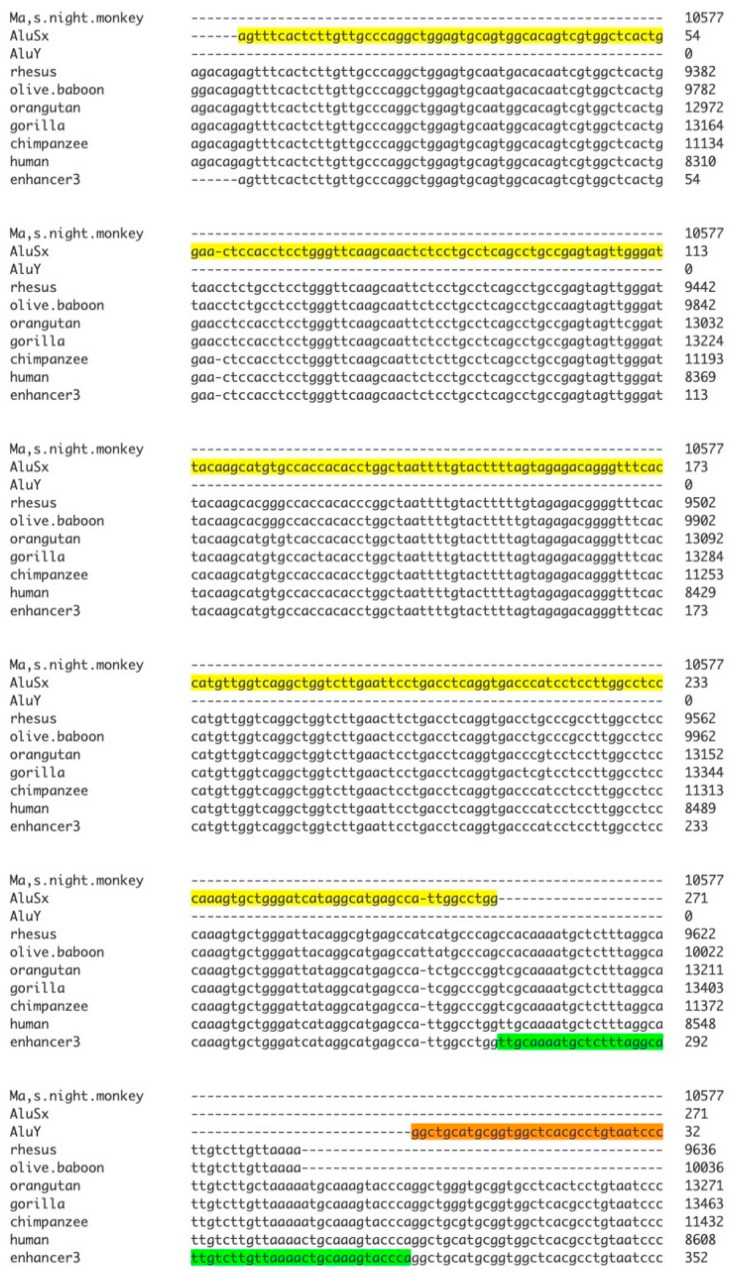
Alignment of homologous primate species sequences with that of enhancer 3. Highlighted regions indicate *AluSx* (yellow), *AluY* (brown), and the intervening spacer (green). Note that the alignment with the *AluY* does not show the total alignment.

Enhancer 3 comprises tandem transposable elements (TEs) *and AluY* separated by a 50 bp spacer (Figure 9). To investigate evolutionary origins, *SMIM45* orthologs from various primate species were aligned with the human enhancer 3. Figure 10 demonstrates that the enhancer originated in the Old World monkey lineage, as evidenced by its presence in the rhesus monkey (*Macaca mulatta*) and olive baboon (*Papio nubis*) *SMIM45* loci, while the enhancer sequence was not detected in Ma’s night monkey (*Aotus nancymaae*), a New World primate. Both the rhesus monkey and olive baboon diverged from the human lineage approximately 25–30 million years ago. Figure 10 shows that the rhesus and baboon sequences possess the complete *AluSx* sequence along with part of the spacer sequence. The enhancer appears to have originated by the insertion of the *AluSx* TE into the Old World monkey (*Catarrhini*) *SMIM45* locus. A second *Alu*, *AluY*, was inserted in the Great Apes (*Hominoidae*) *SMIM45*, as it is found only in the orangutan and the other Great Apes. Because New World monkeys lack the T-rich (TTTT/A) sequence for *AluSx* insertion (Figure S6), the driving force behind enhancer 3 development appears to have been the emergence of the TTTTA-rich sequence in the Old World monkeys. For reference, Figure S3 provides a diagram of primate phylogeny.

To determine in which species the enhancer 3 sequence was completed, we aligned the chimpanzee sequence with the human enhancer 3 sequence (Figure S7). Twelve point mutations differentiate the human and chimp/bonobo sequences from enhancer 3; thus, the enhancer 3 sequence is human-specific. Enhancer 3 contains the sequence TAATTTTGT, which represents the transcription factor NANOG core binding motif consisting of TAAT followed by several Ts and GT [38]. This sequence is carried by *AluSx.*

## 3. Discussion

This study investigates the evolutionary origins of regulatory sequences within the *SMIM45* locus, providing insights into the mechanisms underlying the formation of silencers and enhancers. Notably, we show that, over a massive time span of >400 My, *SMIM45* evolved a suite of regulatory elements believed to control the human-specific 107 aa protein’s activity during human fetal brain development. The complexity of these combined regulatory processes that are vital for human embryonic brain development may explain this long evolutionary time frame. The exonic silencer sequence, which overlaps the 68 aa C-terminal end, is the first of the regulatory sequences to originate evolutionarily, featuring a small number of anciently formed invariant wobble bases; notably, this invariance reflects the silencer’s functional constraints. While the mechanism of fixation of these bases is unknown, the majority of exonic silencer bases are fixed by the cultivator model described by Lee et al. [21]. The data show that the cultivator-fixed bases and the wobble-invariant bases carry information that guides exonic silencer maturation via nearest-neighbor bias, resulting in the total completion of the 38 base pair exonic silencer. De novo origination of both silencer b (segment of the *LOC130067579* ATAC-STARR-seq lymphoblastoid region 13815 in the promoter region) and the 107 aa protein cistron occurred in the Afrotheria clade [7]. Concurrently, the 68 aa C-terminal base sequence was also completed in Afrotheria, hinting at a potential role of these bases (that also make up the exonic silencer-specific fixed bases) in the emergence of silencer b. Pang and Snyder reported that a large number of silencers are found in exons of genes [24]; however, our literature review yielded no existing research on the presence of exonic silencers that partially overlap ORFs, with the exception of our previous study [7]. Located within the *SMIM45* gene, the *LOC130067579* ATAC-STARR-seq lymphoblastoid silent region 13815 appears to be a novel regulatory element and the first of its kind, forming through three intricate processes.

Pu et al. [39] provided a detailed study on the evolution of enhancers containing embedded silencers. Analyzing expression patterns across *Drosophila melanogaster* and related species, they demonstrated that the evolution of repressor sequences can precede that of the enhancer sequences. Although the embedded silencer in the enhancer 2 sequence reported here was completed before the enhancer, the significance of this remains uncertain due to the short evolutionary time span.

We have demonstrated that bias in nearest neighbors of invariant bases in the exonic silencer raises the GC content of the locus to 73% during evolution. Similarly, there is evolutionary pressure to increase GC content within silencer b, which is in the promoter of the 107 aa cistron. High GC content facilitates chromatin accessibility, enabling the transcriptional apparatus to access DNA [40,41] and potentially mediating suppression. We also demonstrate evolutionary pressure to add the CpG motif to the silencer *LOC130067579* sequence. There is a known role of high CpG content in gene repression [18]. Yang et al. [42] confirmed that CpG content impacts gene expression evolution and silencers are recognized for repressing somatic gene expression [43]. The presence of a high GC and CpG content in *LOC130067579* is consistent with the concept that this silencer suppresses the 107 aa cistron in somatic tissues.

The completion of all three enhancer sequences in the human *SMIM45* locus points to the regulation of the human 107 aa cistron, especially given that the orthologous chimpanzee sequence encodes a truncated protein [7]. Concerning *SMIM45* regulatory element functions, a comparison with known related silencers and enhancers is instructive. Although it is not a bicistronic gene, *FGF18* (fibroblast growth factor 18) is similar to *SMIM45* in having super-silencers and super-enhancers [44]. Two silencers in the *FGF18* gene can act together through compensatory chromatin interactions, and compensatory chromatin interactions potentially also relate to the function of the two silencers in *SMIM45*: silent region_13814 and silent region 13815.

Both enhancer 1 and enhancer 2 originated de novo from ancestral non-coding regions and appear to be part of a large de novo class of human-brain-specific enhancers [45]. These brain-specific enhancer sequences are completed by one or a small number of point mutations distinguishing human from chimpanzee sequences. Consistent with this, enhancer 1 and enhancer 2 show a small number of mutations that complete the human sequence. De novo enhancers can induce genes that are critical to cognitive function and are expressed in progenitor cells of the developing neocortex [45]. By analogy, enhancers 1 and 2 may regulate the expression of the *SMIM45* 107 aa protein shown to be produced in the embryonic cerebral cortex [8].

Enhancer 3, the LOC127896430 NANOG hESC enhancer, consists of two *Alu* elements and contains the transcription factor NANOG core binding motif, which is known to bind enhancers in embryonic stem cells to regulate gene expression during developmental processes [30,46]. This may relate to the regulation of the 107 aa cistron by enhancer 3, as the 107 aa protein is expressed in embryonic brain tissues [8].

The aforementioned analogies provide important insights as to how these enhancers and silencers may activate or suppress the 107 aa cistron and remain promising targets for future studies.

## 4. Conclusions

*SMIM45* appears to represent a unique bicistronic gene characterized by an intricate suite of regulatory sequences. Evolving over several hundred million years, these transcriptional enhancers and silencers emerged through complex, distinct processes. Silencer *LOC130067579*, ATACSTARR-seq lymphoblastoid silent region 13815, consists of two segments: one overlapping an ORF (exonic silencer) and the other adjacent to the 107 aa promoter (silencer b). These two segments developed independently across different evolutionary time frames. The exonic silencer formed through diverse processes and was the first regulatory sequence to originate during evolution of *SMIM45*; importantly, it appears to be a novel regulatory element. The results point to a complex regulation of the de novo 107 aa cistron during human embryonic brain development. Notably, analogies with known enhancers and silencers suggest how the *SMIM45* enhancers silencers may activate or suppress the 107 aa cistron in both somatic and embryonic tissues.

## 5. Methods

### 5.1. Source of Properties of Human SMIM45

The NCBI Gene database (https://www.ncbi.nlm.nih.gov/gene, accessed on 30 December 2025) is the source for *SMIM45* properties, including enhancers, silencers and their experimental evidence, accessible chromatin regions, functions, sequences, and RNA transcript exon/intron sequences.

### 5.2. SMIM45 in Other Species

The *SMIM45* orthologous sequences from various species were also obtained from the NCBI Gene database (https://www.ncbi.nlm.nih.gov/gene, accessed on 17 December 2025), except for that of the elephant shark. The elephant shark *SMIM45* sequence is available from the following website:

*Ensembl* (Ensembl genome brower 115)*:* http://may2025.archive.ensembl.org/ (accessed on 20 December 2025) Alignment Methods. For species where *SMIM45* has not been annotated, the total sequence between flanking genes *CENPM* and *SEPTIN 3* was used for alignments.

### 5.3. Nucleotide and Amino Acid Sequence Alignments

Clustal Omega multiple sequence alignment, version 1.2 and pairwise sequence alignment EMBOSS Needle Tools (EMBL-EBI) [47] were used.

Several sequences were aligned using MAFFT (version 7) [48] (https://mafft.cbrc.jp/alignment/server/index.html, accessed on 15 January 2025). This sequence program allowed for easy visualization of conserved bases.

### 5.4. CpG Pairs in Silencer B (In the Promoter)

There are 13 CpG pairs in silencer b. The probability of finding 13 Cs followed by 13 Gs in a sequence of 192 bases containing 73% G+C is approximately 6.956 × 10^−10^ (determined via Google statistical analysis). This supports the statement of a high CpG content present in silencer b.

### 5.5. Sequences of Silencer LOC130067579 ATAC-STARR-seq Lymphoblastoid Silent Region 13815 and Its Segments

Silencer *LOC130067579*

tgcgcgacaacctggccttcggcggcccggaggtctgagccgacttgcaaaggggatagg

cgggcggcaccgggcgccctcccccagcccgccccgcccgcccagcccggagacccccaa

ggcagagggaggccggcctgttggccctccacgctatccctctgcagcctgggccctccc

gacagaggccccaggtgcgctggcagtggaggtggggcacttaggtgcct 230 bps

exonic silencer (overlap with 68 aa)

tgcgcgacaacctggccttcggcggcccggaggtctga 38 bps

silencer b (in the promoter)

gccgacttgcaaaggggataggcgggcggcaccgggcgccctcccccagcccgccccgcc

cgcccagcccggagacccccaaggcagagggaggccggcctgttggccctccacgctatc

cctctgcagcctgggccctcccgacagaggccccaggtgcgctggcagtggaggtggggc

acttaggtgcct 192 bps

*Enhancer sequences*

Enhancer 1, *LOC130067578* ATAC-STARR-seq lymphoblastoid active region 19151 [*Homo sapiens* (human)] Gene ID: 130067578 ctagtggctgaagcaccgcccaggaggaaaaaccggcgggggaagcagggccgcctgcacctaccaagatggtggccgtgttcaggccgggcagcttgtccaggggcctcaacaccgacatcacagccgcaggaccaaccgttgctcctgcggtgcgcgccgatctttcaaaccgccctgagtccagcccctagagcgcggcctgggggc 210 bps

Enhancer 2 *LOC127896429* H3K4me1 hESC enhancer GRCh37_chr22:42346983-42347610 [*Homo sapiens* (human)] Gene ID: 127896429

gagactccgtctcaaaaaaaacaaaccctctgtgaactcacagtcaccccccagtcccacatatgctggaaaggacctgtcatacctgaagagcccctagatggcgcagaggtgtctgtggtgggggacctaggtcctgaagccacctcacccagaggctttccccctgcccatccccaggtttctgggaacggattccctagggaggtggttcctggaagccttttcccagccacgccccgtgggccctagggggctgctctctccctcctgagaatagccctcaacacgtggcagataccttgtctatggcatagggggagggggaggatccatgcttgggaaggtggaccccacccccaacgtcagctcttggctttgaattccagctcagtcactgagaagctgagggctctgggagaaggagaaggccagcagcatcacctctctgcctcatcccaaaatggggtctcaacaccaatccagctgggaggactgcaggaagtgatgttggggccagctggaagatgggagtgctcaatgcctgtgctggctgtacaccagccaggggtgctgtggggtagatgaggcagaatggggagggggagccatttgcaagggtcctgaa 628 bps

Embedded silencer in LOC127896429 (silent region_13814)

ctagatggcgcagaggtgtctgtggtgggggacctaggtcctgaagccacctcacccaga 60 bps

Enhancer 3 LOC127896430 NANOG hESC enhancer GRCh37_chr22:42351209-42351720 [Homo sapiens (human)] Gene ID: 127896430agtttcactcttgttgcccaggctggagtgcagtggcacagtcgtggctcactggaactccacctcctgggttcaagcaactctcctgcctcagcctgccgagtagttgggattacaagcatgtgccaccacacctggctaattttgtacttttagtagagacagggtttcaccatgttggtcaggctggtcttgaattcctgacctcaggtgacccatcctccttggcctcccaaagtgctgggatcataggcatgagccattggcctggttgcaaaatgctctttaggcattgtcttgttaaaactgcaaagtacccaggctgcatgcggtggctcacgcctgtaatcccagcactttgggaggccgaggtgggcggatcacgaggtcaggagatcaagaccatcctggctaacacggtgaaaccccgtctctactaaaaatacaaaaaattagctgggtgcagtggcggtcacctgcagtcccagctactcaggaggctgaggcaggag 512 bps

### 5.6. Determinations of Alus in Enhancer 3

RepeatMasker Current Version: open-4.0.9 ( Dfam: 3.0 only*) [37] was utilized for the determination of *Alu* transposable elements and their locations in *SMIM45.*

### 5.7. Translation of Nucleotide Sequence

The ExPASy Translational tool (website: https://web.expasy.org/translate/, accessed on 15 December 2025) [49] was employed to translate the 68 aa C-terminal end nucleotide sequence.

## Figures and Tables

**Table 1:** Properties of enhancers related to enhancers 1–3.

Enhancer	General Properties	Reference
Enhancer 1 *LOC130067578* ATAC-STARR-seq Lymphoblastoid active region 19151	Binds transcription factor activators and silencers within chromatin-accessible regions	[22]
Enhancer 2 LOC127896429 H3K4me1 hESC enhancer	Active in embryonic stem cells and associated with activation of gene expression	[15]
Enhancer 2 *LOC127896429* embedded silencer, silent region_13814	Binds repressor transcription factors and prevents transcription; essential to secure gene expression in specific tissues at specific times (spatiotemporally).	[28]
Enhancer 3 *LOC127896430 NANOG* hESC enhancer	*Alus* act as enhancers and can regulate nearby promoters; NANOG is a regulator of human embryonic stem cell (hESC) identity	[29,30]

**Table 2:** Nearest-neighbor bases of invariant bases.

Invariant Base	5′ of Invariant Base
C3	G2
G6	C5
A9	C8
T13	C12
T18	C17
C26	G25
T34	G33
**Invariant Base**	**3′ of Invariant Base**
A10	C11
G22	T23
G15	C16
C20	G21
C28	G29
A31	G32

## Data Availability

All data are in the manuscript and Supplementary Materials.

## Supplementary Materials

The following supporting information can be downloaded at https://www.mdpi.com/article/10.3390/genes17050519/s1.

## Abbreviations

Open reading frame, ORF; internal ribosome entry site, IRES; upstream open reading frame, uORF; amino acid, aa; transcription factor, TF; transposable element, TE; multiple sequence alignment, MSA.

## References

[1] Gray T.A., Saitoh S., Nicholls R.D. (1999). An imprinted, mammalian bicistronic transcript encodes two independent proteins. Proc. Natl. Acad. Sci. USA.

[2] Lu Y., Zhang Y., Hang X., Qu W., Lubec G., Chen C., Zhang C. (2013). Genome-wide computational identification of bicistronic mRNA in humans. Amino Acids.

[3] Wu B., Cox M.P. (2021). Characterization of Bicistronic Transcription in Budding Yeast. mSystems.

[4] Park J., Farris S. (2021). Spatiotemporal Regulation of Transcript Isoform Expression in the Hippocampus. Front. Mol. Neurosci..

[5] Dueñas M.A., Craig R.J., Gallaher S.D., Moseley J.L., Merchant S.S. (2025). Leaky ribosomal scanning enables tunable translation of bicistronic ORFs in green algae. Proc. Natl. Acad. Sci. USA.

[6] Alghoul F., Laure S., Eriani G., Martin F. (2021). Translation inhibitory elements from Hoxa3 and Hoxa11 mRNAs use uORFs for translation inhibition. eLife.

[7] Delihas N. (2024). Evolution of a Human-Specific De Novo Open Reading Frame and Its Linked Transcriptional Silencer. Int. J. Mol. Sci..

[8] Xiao C., Mo F., Lu Y., Xiao Q., Yao C., Li T., Qi J., Liu X., Chen J.-Y., Zhang L. (2024). Reply to: Identification of old coding regions disproves the hominoid de novo status of genes. Nat. Ecol. Evol..

[9] Xu X., Zhang J., Mo F., Chen J.Y., Delihas N., Zhang L., An N.A., Li C.Y., Liu X., Xiao C. (2024). Origin of functional de novo genes in humans from “hopeful monsters”. Wiley Interdiscip. Rev. RNA.

[10] Raj A., Wang S.H., Shim H., Harpak A., Li Y.I., Engelmann B., Stephens M., Gilad Y., Pritchard J.K. (2016). Thousands of novel translated open reading frames in humans inferred by ribosome footprint profiling. eLife.

[11] Pennacchio L.A., Bickmore W., Dean A., Nobrega M.A., Bejerano G. (2013). Enhancers: Five essential questions. Nat. Rev. Genet..

[12] Levine M. (2010). Transcriptional enhancers in animal development and evolution. Curr. Biol..

[13] Maurya S.S. (2021). Role of Enhancers in Development and Diseases. Epigenomes.

[14] Hnisz D., Abraham B.J., Lee T.I., Lau A., Saint-André V., Sigova A.A., Hoke H.A., Young R.A. (2013). Super-Enhancers in the Control of Cell Identity and Disease. Cell.

[15] Whyte W.A., Orlando D.A., Hnisz D., Abraham B.J., Lin C.Y., Kagey M.H., Rahl P.B., Lee T.I., Young R.A. (2013). Master transcription factors and mediator establish super-enhancers at key cell identity genes. Cell.

[16] Ko J.Y., Oh S., Yoo K.H. (2017). Functional Enhancers As Master Regulators of Tissue-Specific Gene Regulation and Cancer Development. Mol. Cells.

[17] Moorthy S.D., Davidson S., Shchuka V.M., Singh G., Malek-Gilani N., Langroudi L., Martchenko A., So V., Macpherson N.N., Mitchell J.A. (2017). Enhancers and super-enhancers have an equivalent regulatory role in embryonic stem cells through regulation of single or multiple genes. Genome Res..

[18] Segert J.A., Gisselbrecht S.S., Bulyk M.L. (2021). Transcriptional Silencers: Driving Gene Expression with the Brakes On. Trends Genet..

[19] Huang D., Petrykowska H.M., Kumar D., Kardava L., Moir S., Afzali B., Elnitski L., Ovcharenko I. (2025). Super-silencers are crucial for development and carcinogenesis in B cells. Nat. Commun..

[20] Emera D., Yin J., Reilly S.K., Gockley J., Noonan J.P. (2016). Origin and evolution of developmental enhancers in the mammalian neocortex. Proc. Natl. Acad. Sci. USA.

[21] Lee U., Mozeika S.M., Zhao L. (2024). A Synergistic, Cultivator Model of De Novo Gene Origination. Genome Biol. Evol..

[22] Hansen T.J., Hodges E. (2022). ATAC-STARR-seq reveals transcription factor-bound activators and silencers within chromatin-accessible regions of the human genome. Genome Res..

[23] Ahituv N. (2016). Exonic enhancers: Proceed with caution in exome and genome sequencing studies. Genome Med..

[24] Pang B., Snyder M.P. (2020). Systematic identification of silencers in human cells. Nat. Genet..

[25] An N.A., Zhang J., Mo F., Luan X., Tian L., Shen Q.S., Li X., Li C., Zhou F., Zhang B. (2023). De novo genes with an lncRNA origin encode unique human brain developmental functionality. Nat. Ecol. Evol..

[26] Davis A.A., Temple S. (1994). A self-renewing multipotential stem cell in embryonic rat cerebral cortex. Nature.

[27] Boyer L.A., Lee T.I., Cole M.F., Johnstone S.E., Levine S.S., Zucker J.P., Guenther M.G., Kumar R.M., Murray H.L., Jenner R.G. (2005). Core transcriptional regulatory circuitry in human embryonic stem cells. Cell.

[28] Zeitlinger J. (2020). Seven myths of how transcription factors read the cis-regulatory code. Curr. Opin. Syst. Biol..

[29] Liang L., Cao C., Ji L., Cai Z., Wang D., Ye R., Chen J., Yu X., Zhou J., Bai Z. (2023). Complementary Alu sequences mediate enhancer-promoter selectivity. Nature.

[30] Yan J., Luo R., Rosen B.P., Liu D., Wong W., Leslie C.S., Huangfu D. (2025). Discovery of NANOG enhancers and their essential roles in self-renewal and differentiation in human embryonic stem cells. Stem Cell Rep..

[31] Arndt P.F., Burge C.B., Hwa T. (2003). DNA sequence evolution with neighbor-dependent mutation. J. Comput. Biol..

[32] Branco M.R., Chuong E.B. (2020). Crossroads between transposons and gene regulation. Philos. Trans. R. Soc. Lond. B Biol. Sci..

[33] Häsler J., Strub K. (2006). Alu elements as regulators of gene expression. Nucleic Acids Res..

[34] Su M., Han D., Boyd-Kirkup J., Yu X., Liang L., Han J.J. (2014). Evolution of Alu elements toward enhancers. Cell Rep..

[35] Chen L.L., Yang L. (2017). ALUternative Regulation for Gene Expression Trends. Trends Cell Biol..

[36] Borsari B., Villegas-Mirón P., Pérez-Lluch S., Turpin I., Laayouni H., Segarra-Casas A., Bertranpetit J., Guigó R., Acosta S. (2021). Enhancers with tissue-specific activity are enriched in intronic regions. Genome Res..

[37] Storer J., Hubley R., Rosen J., Wheeler T.J., Smit A.F. (2021). The Dfam community resource of transposable element families, sequence models, and genome annotations. Mob. DNA.

[38] Jauch R., Ng C.K., Saikatendu K.S., Stevens R.C., Kolatkar P.R. (2008). Crystal structure and DNA binding of the homeodomain of the stem cell transcription factor Nanog. J. Mol. Biol..

[39] Pu J., Wang Z., Cong H., Chin J.S.R., Justen J., Finet C., Yew J.Y., Chung H. (2021). Repression precedes independent evolutionary gains of a highly specific gene expression pattern. Cell Rep..

[40] Dekker J. (2007). GC- and AT-rich chromatin domains differ in conformation and histone modification status and are differentially modulated by Rpd3p. Genome Biol..

[41] Fenouil R., Cauchy P., Koch F., Descostes N., Cabeza J.Z., Innocenti C., Ferrier P., Spicuglia S., Gut M., Gut I. (2012). CpG islands and GC content dictate nucleosome depletion in a transcription-independent manner at mammalian promoters. Genome Res..

[42] Yang H., Li D., Cheng C. (2014). Relating gene expression evolution with CpG content changes. BMC Genom..

[43] Zhu X., Huang L., Wang C., Li G., Deng B., Kong D., Wang X., Chang R., Gu Y., Wen Q. (2025). Uncovering the whole genome silencers of human cells via Ss-STARR-seq. Nat. Commun..

[44] Zhang Y., Chen K., Tang S.C., Cai Y., Nambu A., See Y.X., Fu C., Raju A., Lebeau B., Ling Z. (2025). Super-silencer perturbation by EZH2 and REST inhibition leads to large loss of chromatin interactions and reduction in cancer growth. Nat. Struct. Mol. Biol..

[45] Li S., Hannenhalli S., Ovcharenko I. (2023). De novo human brain enhancers created by single-nucleotide mutations. Sci. Adv..

[46] Heurtier V., Owens N., Gonzalez I., Mueller F., Proux C., Mornico D., Clerc P., Dubois A., Navarro P. (2019). The molecular logic of Nanog-induced self-renewal in mouse embryonic stem cells. Nat. Commun..

[47] Madeira F., Madhusoodanan N., Lee J., Eusebi A., Niewielska A., Tivey A.R.N., Lopez R., Butcher S. (2024). The EMBL-EBI Job Dispatcher sequence analysis tools framework in 2024. Nucleic Acids Res..

[48] Katoh K., Rozewicki J., Yamada K.D. (2019). MAFFT online service: Multiple sequence alignment, interactive sequence choice and visualization. Brief. Bioinform..

[49] Gasteiger E., Gattiker A., Hoogland C., Ivanyi I., Appel R.D., Bairoch A. (2003). ExPASy: The proteomics server for in-depth protein knowledge and analysis. Nucleic Acids Res..

