# Supplementary material for: Diverse Processes Drive the Origination and Maturation of an Array of Enhancers and Silencers During a Vast Evolutionary Timescale of a Bicistronic Gene
Source: Genes (Basel). 2026 Apr 28;17(5):519. doi: 10.3390/genes17050519 (PMC13205175; doi:10.3390/genes17050519)
Supplement: Supplementary file 1 [file genes-17-00519-s001.zip › genes-4250630-supplementary.pdf]

Supplementary File S1

Diverse processes drive the origination and maturation an Array of Enhancers and Silencers during a vast evolutionary timescale of the bicistronic gene

Nicholas Delihas
Department of Microbiology and Immunology
Renaissance School of Medicine; Stony Brook University; Stony Brook, NY 11794 USA
Correspondence: Nicholas.delihas@stonybrook.edu; Tel. # 001 631 286-9427
Orchid https://orcid.org/0000-0002-1704-2587

Figure S1. Alignment of human silencer b with homologous sequences from the gorilla and chimpanzee. While the chimpanzee *SMIM45* gene contains a silencer b sequence identical to that of humans, both species show three point mutations compared with the homologous gorilla sequence. Clustal Omega, Multiple Sequence Alignment was used for alignment.

```
CLUSTAL O(1.2.4) multiple sequence alignment


gorilla              gccgacttgcaaaggggataggcgggcggcaccgggcgccctcccccagtccgccccgcc     60
chimpanzee           gccgacttgcaaaggggataggcgggcggcaccgggcgccctcccccagcccgccccgcc     60
human.silencer.b     gccgacttgcaaaggggataggcgggcggcaccgggcgccctcccccagcccgccccgcc     60
                     ************************************************* **********

gorilla              cgcccagcccggagacccccaaggcagagggaggcctgcctcttggccctccacgctatc     120
chimpanzee           cgcccagcccggagacccccaaggcagagggaggccggcctgttggccctccacgctatc     120
human.silencer.b     cgcccagcccggagacccccaaggcagagggaggccggcctgttggccctccacgctatc     120
                     ************************************ **** ******************

gorilla              cctctgcagcctgggccctcgcgacagaggccccaggtgcgctggcagtggaggtggggc     180
chimpanzee           cctctgcagcctgggccctcccgacagaggccccaggtgcgctggcagtggaggtggggc     180
human.silencer.b     cctctgcagcctgggccctcccgacagaggccccaggtgcgctggcagtggaggtggggc     180
                     ******************** ***************************************

gorilla              acttaggtgcctggctggcccagggcttg     209
chimpanzee           acttaggtgcct-----------------     192
human.silencer.b     acttaggtgcct-----------------     192
                     ************
```

FigureS2. Alignment diagram shows the GC sequence addition in the Great Apes, except for that of the orangutan. GC addition in alignment silencer b with species of the Afrothere and primates. MAFFT (https://mafft.cbrc.jp/alignment/server/index.html) was used for alignment.

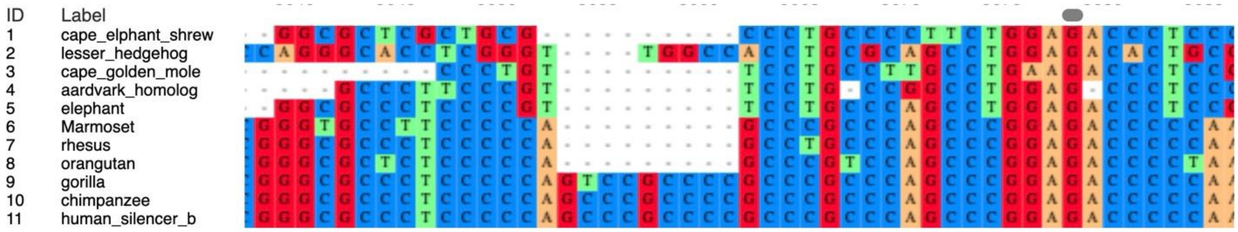

Figure S3. Diagram of the primate phylogenetic tree. Drawing is from Wikimedia Commons and is in the public domain.

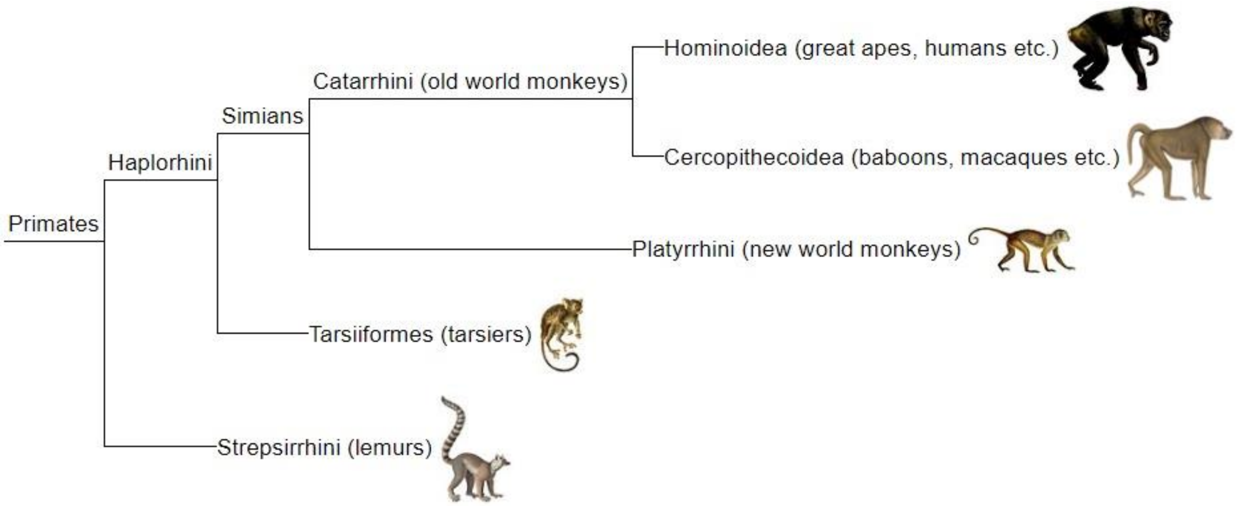

Figure S4. The alignment of the chimpanzee sequence homologous to enhancer 2 with the *SMIM45* enhancer 2. Alignment shows two bp deletions and three point mutations that distinguish the human sequence from that of the chimpanzee. Clustal Omega, Multiple Sequence Alignment was used for alignment.

```
chimpanzee      ccagcctggcgaacacagcgagactccgtctcaaaaaaaacaaaccctctgtgaactcac 6900
enhancer2       -------------------gagactccgtctcaaaaaaaacaaaccctctgtgaactcac 41
                                   *****************************************

chimpanzee      agtcaccccccagtcccacatatgctggaaaggacccgtcatacctgaagagcccctaga 6960
enhancer2       agtcaccccccagtcccacatatgctggaaaggacctgtcatacctgaagagcccctaga 101
                ************************************ ***********************

chimpanzee      tggcgcagaggtgtctgtggtgggggacctaggtcctgaagccacctcacccagaggctt 7020
enhancer2       tggcgcagaggtgtctgtggtgggggacctaggtcctgaagccacctcacccagaggctt 161
                ************************************************************

chimpanzee      tccccctgcccatccccaggtttctgggaacggattccctagggaggtggttcctggaag 7080
enhancer2       tccccctgcccatccccaggtttctgggaacggattccctagggaggtggttcctggaag 221
                ************************************************************

chimpanzee      cctcttcccagccacgccctgtgggccctagggggctgctctctccctcctgagacaata 7140
enhancer2       ccttttcccagccacgccccgtgggccctagggggctgctctctccctcctga--gaata 279
                *** *************** *********************************   ****

chimpanzee      gccctcaacacgtggcagataccttgtctatggcatagggggagggggaggatccatgct 7200
enhancer2       gccctcaacacgtggcagataccttgtctatggcatagggggagggggaggatccatgct 339
                ************************************************************

chimpanzee      tgggaaggtggaccccacccccaacgtcagctcttggctttgaattccagctcagtcact 7260
enhancer2       tgggaaggtggaccccacccccaacgtcagctcttggctttgaattccagctcagtcact 399
                ************************************************************

chimpanzee      gagaagctgagggctctgggagaaggagaaggccagcagcatcacctctctgcctcatcc 7320
enhancer2       gagaagctgagggctctgggagaaggagaaggccagcagcatcacctctctgcctcatcc 459
                ************************************************************

chimpanzee      caaaatggggtctcaacaccaatccagctgggaggactgcaggaagtgatgttggggcca 7380
enhancer2       caaaatggggtctcaacaccaatccagctgggaggactgcaggaagtgatgttggggcca 519
                ************************************************************

chimpanzee      gctggaagatgggagtgctcaatgcctgtgctggctgtacaccagccaggggtgctgtgg 7440
enhancer2       gctggaagatgggagtgctcaatgcctgtgctggctgtacaccagccaggggtgctgtgg 579
                ************************************************************

chimpanzee      ggtagatgaggcagaatggggagggggagccatttgcaagggtcctgaaagccaggctag 7500
enhancer2       ggtagatgaggcagaatggggagggggagccatttgcaagggtcctgaa----------- 628
                *************************************************
```

Figure S5. The evolutionary completion of the embedded silencer in the chimpanzee. . Alignment of Great Apes sequences homologous to the human embedded silencer in enhancer 2 sequence. Clustal Omega, Multiple Sequence Alignment was used for alignment.

```
#  Percent Identity  Matrix - created by Clustal2.1

     1: orangutan          100.00   96.17   96.38   96.51   96.39   94.92
     2: gorilla             96.17  100.00   97.83   97.58   97.29   95.00
     3: bonobo              96.38   97.83  100.00  100.00   99.36  100.00
     4: chimpanzee          96.51   97.58  100.00  100.00   99.35  100.00
     5: human.enhancer2     96.39   97.29   99.36   99.35  100.00  100.00
     6: embedded.silencer   94.92   95.00  100.00  100.00  100.00  100.00
```

```
orangutan            agagcccctagacggcccagaggtgtctgtgttgggggacctaggtcctgaagccacctc   9065
gorilla              agagcccctatatggcccagaggtgtctgtggtgggggacctaggtcctgaagctacctc   9027
bonobo               agagcccctagatggcgcagaggtgtctgtggtgggggacctaggtcctgaagccacctc   5008
chimpanzee           agagcccctagatggcgcagaggtgtctgtggtgggggacctaggtcctgaagccacctc   149
human.enhancer2      agagcccctagatggcgcagaggtgtctgtggtgggggacctaggtcctgaagccacctc   149
embedded.silencer    -------ctagatggcgcagaggtgtctgtggtgggggacctaggtcctgaagccacctc   53
                            *** * *** ************** ********************** *****

orangutan            a-ccagaagctttcccccctgcccatccccaggtttctgggaacagattccctagggaggt   9124
gorilla              acccagaggctttcccccctgcccatccccaggtttctgggaacggattccctagggaggt   9087
bonobo               acccagaggctttcccccctgcccatccccaggtttctgggaacggattccctagggaggt   5068
chimpanzee           acccagaggctttcccccctgcccatccccaggtttctgggaacggattccctagggaggt   209
human.enhancer2      acccagaggctttcccccctgcccatccccaggtttctgggaacggattccctagggaggt   209
embedded.silencer    acccaga-----------------------------------------------------   60
                     * *****
```

Figure S6. The T-rich (TTTT/A) motif is present in the genomic region upstream of the enhancer 3/*AluSx* sequence in the Old World monkeys and Great Apes. The motif is absent in the New World monkey genomic region. Alignment of sequences from the New World monkey, Old World monkeys and the Great Apes with the sequences of *AluSx* and enhancer 3.

```
Ma,s.night.monkey              ------------------------------------------------------------   10577
rhesus                         ttctgtgccaggcagaatggtgcaaaatgcttttttattttaa-ttatttcttttttttg   9322
olive.baboon                   ttctgcgccaggcagaatggtgcaaaatgcttttttattttaa-ttatttcttttttttg   9722
orangutan                      ttctgtgccaggcagaatggtgcaaaatgcttttttattttaatttattatttttttttg   12912
gorilla                        ttctgtgccaggcagaatggtgcaaaatgcttttttatttta---tttta--tttttttg   13104
chimpanzee                     ttctgtgccaggcagaatggtgcaaaatgctttttaattttaattttttt--tttttttg   11074
SMIM45.human.seq               ttctgtgccaggcagaatggtgcaaaatgcttttttattttaa-tttttt--tttttttg   8250
Enhancer3.NANOG.hESC.enhancer  ------------------------------------------------------------   0
AluSx                          ------------------------------------------------------------   0

Ma,s.night.monkey              ------------------------------------------------------------   10577
rhesus                         agacagagtttcactcttgttgcccaggctggagtgcaatgacacaatcgtggctcactg   9382
olive.baboon                   ggacagagtttcactcttgttgcccaggctggagtgcaatgacacaatcgtggctcactg   9782
orangutan                      agacagagtttcactcttgttgcccaggctggagtgcaatggcacagtcgtggctcactg   12972
gorilla                        agacagagtttcactcttgttgcccaggctggagtgcaatggcacagtcgtggctcactg   13164
chimpanzee                     agacagagtttcactcttgttgcccaggctggagtgcagtggcacagtcgtggctcactg   11134
SMIM45.human.seq               agacagagtttcactcttgttgcccaggctggagtgcagtggcacagtcgtggctcactg   8310
Enhancer3.NANOG.hESC.enhancer  ------agtttcactcttgttgcccaggctggagtgcagtggcacagtcgtggctcactg   54
AluSx                          ------agtttcactcttgttgcccaggctggagtgcagtggcacagtcgtggctcactg   54
```

Figure S7. Alignment of the sequences of the chimpanzee and bonobo homologous to enhancer 3 with that of *SMIM45* enhancer 3. Alignment shows twelve bp mutations that distinguish the human sequence from that of the chimpanzee and bonobo. Alignment using Clustal Omega, Multiple Sequence Alignment was used for alignment.

```
#
#  Percent Identity  Matrix - created by Clustal2.1
#
#

     1: bonobo                 100.00   99.57   99.04   97.85
     2: chimpanzee              99.57  100.00   98.79   97.46
     3: human                   99.04   98.79  100.00  100.00
     4: enhancer3.NANOG.hESC    97.85   97.46  100.00  100.00


bonobo                 ggtgcaaaatgctttttaattttaatttt-tttttttttgagacagagtttcactcttgt    9107
chimpanzee             ggtgcaaaatgctttttaattttaattttttttttttttgagacagagtttcactcttgt    3094
human                  ggtgcaaaatgcttttttattttaatttt-tttttttttgagacagagtttcactcttgt    8270
enhancer3.NANOG.hESC   ----------------------------------------------agtttcactcttgt    14
                                                                     **************

bonobo                 tgcccaggctggagtgcagtggcacagtcgtggctcactggaactccacctcctgggttc    9167
chimpanzee             tgcccaggctggagtgcagtggcacagtcgtggctcactggaactccacctcctgggttc    3154
human                  tgcccaggctggagtgcagtggcacagtcgtggctcactggaactccacctcctgggttc    8330
enhancer3.NANOG.hESC   tgcccaggctggagtgcagtggcacagtcgtggctcactggaactccacctcctgggttc    74
                       ************************************************************

bonobo                 aagcaattctcttgcctcagcctgccgagtagttgggatcacaagcatgtgccaccacac    9227
chimpanzee             aagcaattctcttgcctcagcctgccgagtagttgggatcacaagcatgtgccaccacac    3214
human                  aagcaactctcctgcctcagcctgccgagtagttgggattacaagcatgtgccaccacac    8390
enhancer3.NANOG.hESC   aagcaactctcctgcctcagcctgccgagtagttgggattacaagcatgtgccaccacac    134
                       ****** **** *************************** ********************

bonobo                 ctggctaattttgtacttttagtagagacagggtttcaccatgttggtcaggctggtctt    9287
chimpanzee             ctggctaattttgtacttttagtagagacagggtttcaccatgttggtcaggctggtctt    3274
human                  ctggctaattttgtacttttagtagagacagggtttcaccatgttggtcaggctggtctt    8450
enhancer3.NANOG.hESC   ctggctaattttgtacttttagtagagacagggtttcaccatgttggtcaggctggtctt    194
                       ************************************************************

bonobo                 gaactcctgacctcaggtgacccatcctccttggcctcccaaagtgctgggattataggc    9347
chimpanzee             gaactcctgacctcaggtgacccatcctccttggcctcccaaagtgctgggattataggc    3334
human                  gaattcctgacctcaggtgacccatcctccttggcctcccaaagtgctgggatcataggc    8510
enhancer3.NANOG.hESC   gaattcctgacctcaggtgacccatcctccttggcctcccaaagtgctgggatcataggc    254
                       *** ************************************************* ******

bonobo                 atgagccattggcccggtcgcaaaatgctctttaggcattgtcttgttaaaatgcaaag    9407
chimpanzee             atgagccattggcccggtcgcaaaatgctctttaggcattgtcttgttaaaatgcaaag    3394
human                  atgagccattggcctggttgcaaaatgctctttaggcattgtcttgttaaactgcaaag    8570
enhancer3.NANOG.hESC   atgagccattggcctggttgcaaaatgctctttaggcattgtcttgttaaactgcaaag    314
                       ************** *** ********************************* *******

bonobo                 tacccaggctgcgtgcggtggctcacgcctgtaatcccagcactttgggaggccgaggcg    9467
chimpanzee             tacccaggctgcgtgcggtggctcacgcctgtaatcccagcactttgggaggccgaggcg    3454
human                  tacccaggctgcatgcggtggctcacgcctgtaatcccagcactttgggaggccgaggtg    8630
enhancer3.NANOG.hESC   tacccaggctgcatgcggtggctcacgcctgtaatcccagcactttgggaggccgaggtg    374
                       ************ ********************************************* *

bonobo                 ggcggatcacgaggtcaggagatcaagaccatcctggctaacacggtgaaaccccgtctc    9527
chimpanzee             ggcggatgacgaggtcaggagatcaagaccatcctggctaacacggtgaaaccccgtctc    3514
human                  ggcggatcacgaggtcaggagatcaagaccatcctggctaacacggtgaaaccccgtctc    8690
enhancer3.NANOG.hESC   ggcggatcacgaggtcaggagatcaagaccatcctggctaacacggtgaaaccccgtctc    434
                       ******* ****************************************************

bonobo                 tactaaaatacaaaaaattagctgggcgcagtggcggtcacctgcagtcccagctactc    9587
chimpanzee             tactaaaatacaaaaaattagctaggcgcagtggcggtcacctgcagtcccagctactc    3574
human                  tactaaaatacaaaaaattagctgggtgcagtggcggtcacctgcagtcccagctactc    8750
enhancer3.NANOG.hESC   tactaaaatacaaaaaattagctgggtgcagtggcggtcacctgcagtcccagctactc    494
                       *********************** ** *********************************

bonobo                 aggaggctgaggcaggagaatggtgtgaacctgggaggcggagcttgcagtgagccgaga    9647
chimpanzee             aggaggctgaggcaggagaatggtgtgaacctgggaggcggagcttgcagtgagccgaga    3634
human                  aggaggctgaggcaggagaatggtgtgaacctgggaggcggagcttgcagtgagccgaga    8810
enhancer3.NANOG.hESC   aggaggctgaggcaggag------------------------------------------    512
                       ******************
```
